# The trajectory of life. Decreasing physiological network complexity through changing fractal patterns

**DOI:** 10.3389/fphys.2015.00169

**Published:** 2015-06-02

**Authors:** Joachim P. Sturmberg, Jeanette M. Bennett, Martin Picard, Andrew J. E. Seely

**Affiliations:** ^1^Faculty of Health and Medicine, School of Medicine and Public Health, The University of NewcastleWamberal, NSW, Australia; ^2^Department of Psychology, The University of North Carolina at CharlotteCharlotte, NC, USA; ^3^Center for Mitochondrial and Epigenomic Medicine, Children's Hospital of Philadelphia and the University of PennsylvaniaPhiladelphia, PA, USA; ^4^Thoracic Surgery and Critical Care Medicine, University of Ottawa and Associate Scientist, Ottawa Hospital Research InstituteOttawa, ON, Canada

**Keywords:** aging, heart rate variability, psychoneuroimmunology, inflammation, bioenergetics, mitochondria, physiological networks

## Abstract

In this position paper, we submit a synthesis of theoretical models based on physiology, non-equilibrium thermodynamics, and non-linear time-series analysis. Based on an understanding of the human organism as a system of interconnected complex adaptive systems, we seek to examine the relationship between health, complexity, variability, and entropy production, as it might be useful to help understand aging, and improve care for patients. We observe the trajectory of life is characterized by the growth, plateauing and subsequent loss of adaptive function of organ systems, associated with loss of functioning and coordination of systems. Understanding development and aging requires the examination of interdependence among these organ systems. Increasing evidence suggests network interconnectedness and complexity can be captured/measured/associated with the degree and complexity of healthy biologic rhythm variability (e.g., heart and respiratory rate variability). We review physiological mechanisms linking the omics, arousal/stress systems, immune function, and mitochondrial bioenergetics; highlighting their interdependence in normal physiological function and aging. We argue that aging, known to be characterized by a loss of variability, is manifested at multiple scales, within functional units at the small scale, and reflected by diagnostic features at the larger scale. While still controversial and under investigation, it appears conceivable that the integrity of whole body complexity may be, at least partially, reflected in the degree and variability of intrinsic biologic rhythms, which we believe are related to overall system complexity that may be a defining feature of health and it's loss through aging. Harnessing this information for the development of therapeutic and preventative strategies may hold an opportunity to significantly improve the health of our patients across the trajectory of life.

## Introduction

**Figure 1:**
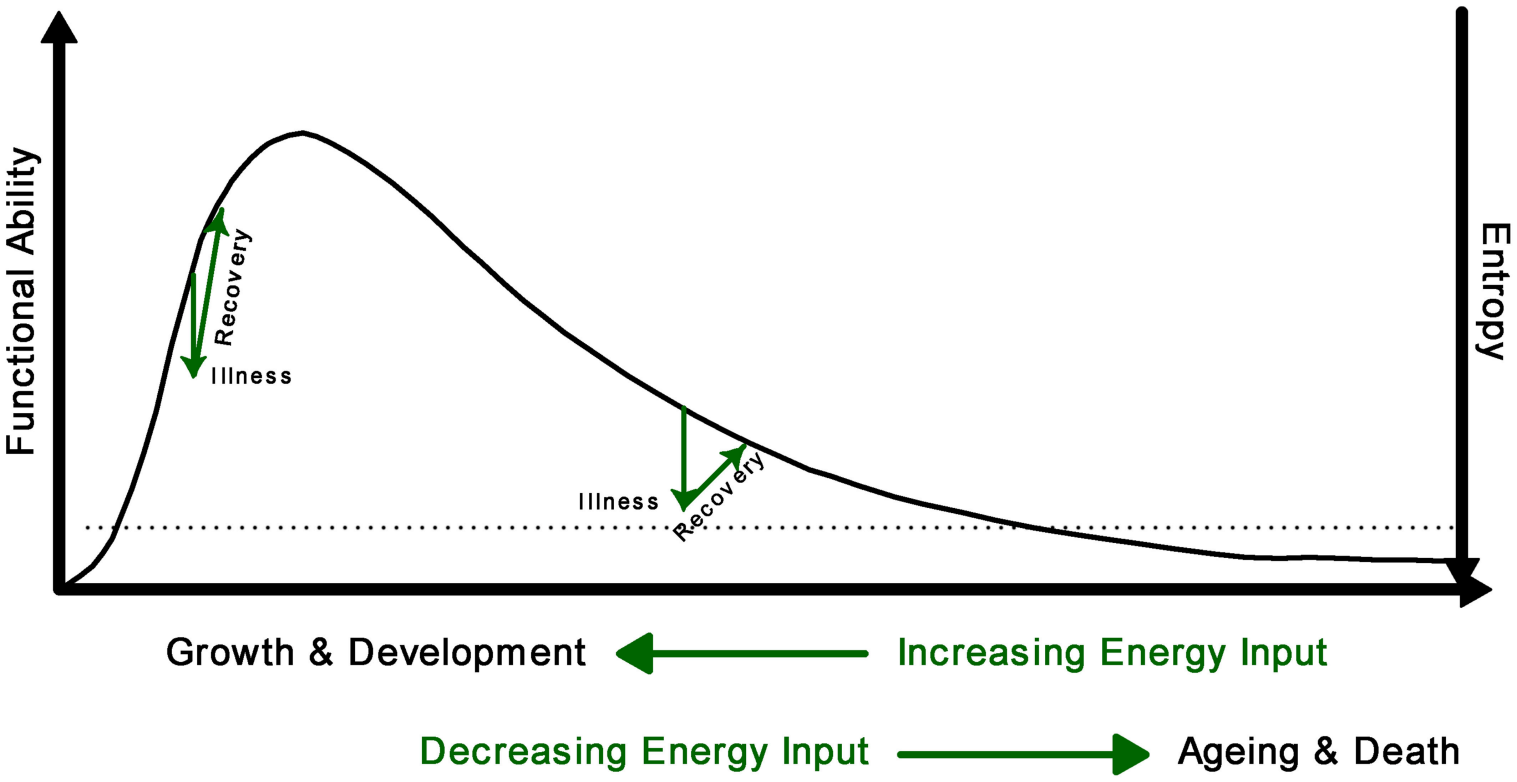
**Conceptual model of the relationship between complexity (functional ability) and entropy across the lifespan**. Illness results in sudden increase of entropy which will be reduced with recuperation. The loss of complexity is associated with an increase in entropy; when entropy reaches the threshold of viability (dotted line), death occurs.

From conception to death, the trajectory of life can be described as a period of growth, plateau, and decline. In this paper, we seek to explore associations between domains of investigation not typically evaluated together to uncover new understanding. Taking a holistic view of the human organism as a non-equilibrium system, we seek to better understand this trajectory by exploring the relationship of physiological complexity and entropy production over time (Figure 1) (Seely and Christou, 2000; Que et al., 2001; Aoki, 2012; Topolski and Sturmberg, 2014). Healthy internal order (negative entropy), including the complex interactions between organ systems, is only made possible if the organism excretes a greater amount of entropy to the environment, which we accomplish largely by burning oxygen. We believe internal order, functional ability and system complexity all increase along with whole body entropy production from conception throughout childhood into early adulthood during the period of growth and development, however there comes a point when physiological complexity, functional ability and entropy productions decrease, at first slowly, and then more abruptly during aging and illness. Illness occurs stochastically, resulting in sharp drops along this curve. Distinct from entropy production (which is the measurable flow of energy through the human organism leading to oxygen consumption and carbon dioxide production), whole body entropy content (total amount of entropy, which is not measurable, inversely related to order and healthy function) decreases through growth (Aoki, 2012), and increases through aging, until rising entropy ultimately becomes incompatible with life and death ensures (Hayflick, 2000; Lipsitz, 2002). Further discussion regarding the multiple definitions of entropy and entropy production may be found here (Seely and Macklem, 2012).

The *loss of complexity hypothesis of aging* postulates that aging is associated with loss of dynamic range or variability in physiological functions; resulting from either the loss or impairment of functional components and/or their non-linear coupling (Lipsitz and Goldberger, 1992; Manor and Lipsitz, 2013). In general terms, this loss of physiological variability results in a reduced adaptive capacity (Lipsitz and Goldberger, 1992; Manor and Lipsitz, 2013). In particular, the loss of complexity in resting dynamics has been postulated to impair reactive adaptive responses (Lipsitz, 2002). However, adaptation is also affected by task demands and/or environmental constraints which have shown bidirectional changes in complexity in relation to manual tasks (Manor and Lipsitz, 2013). Healthy behavior and function of the organism therefore depends upon physiological inter-dependent dynamic interactions among organ systems, leading to highly complex variations that reflect a healthy adaptive system.

Consequently health and disease can no longer be simply defined in dichotomous terms—present or absent; it is continuously redefined as people adapt to changing functional abilities throughout the spectrum of life. Health in subjective and objective terms occurs within a *homeostatic/homeokinetic range* that allows “normal function” on a day-to-day basis (Que et al., 2001; Nicolini et al., 2012). In physiological terms the homeostatic/homeokinetic range may be characterized by organ system variability characteristics. Variability is defined as patterns of variation over intervals-in-time, measured by numerous techniques all calculating attributes to the degree and character of variation (e.g., time- and frequency-domain techniques, detrended fluctuation analysis, sample entropy analysis, or standard deviation, to name a few). Remarkably, biologic variation demonstrates multi-scale self-similar correlation (i.e., fractal patterns), measured with a scaling index whose slope α ranges between >0.5 and <1.0 (Kaplan et al., 1991). In addition to fractal correlation, loss of degree or complexity of variability of a smaller or larger degree usually defines phenotypical patterns of disease as initially described by Goldberger and West in relation to heart rate variability (Goldberger and West, 1987; Goldberger, 1996). However, one limitation of these studies is the lack of understanding the temporal relationships between the normal and abnormal variability patterns throughout the trajectory of life as described in Figure 1, and how such understanding could help in managing patients in a variety of clinical scenarios in a more timely fashion. This is a particular concern in relation to the physiological variability characteristics near the end of life's trajectory when illness may occur involving more than a single organ “malfunction.”

It is noteworthy that words such as variability, complexity, fractals, and entropy are confusing and merit clear definitions. Here we define variability analysis as all measures that reflect degree and character of variation over intervals in time. We distinguish between system complexity, which remains a still poorly defined concept that we simply hypothesize is connected to health, and time-series complexity, which is measured by several mathematical measures of the degree of irregularity and information as well as its fractal characteristics contained in a time series (e.g., inter-beat interval time-series measured continuously for 5 min). Fractals are scale-free self-similar structures in either time or space. For example, as discussed below, healthy heart rate variability is found to be increased in degree, highly complex, and contains fractal properties; all are measured using separate variability analysis metrics. Entropy production is the entropy (heat production divided by temperature) produced per unit time by the human organism, which is required to sustain vibrant internal order and health.

In this contribution to the special theme edition of *Frontiers in Physiology* we allude to the various network functions of the human organism by (1) discussion of the “omics,” arousal/stress systems and immune function, and bioenergetics, (2) show their key interconnections, and (3) suggest that aging is characterized by a loss of variability within functional units at the small scale which are reflected by diagnostic features at the larger scale. Based on these insights we propose an approach to explore the temporal patterns of aging changes through heart and respiratory rate variability (HRV and RRV), gait variability, neuroendocrine-immune communication, and mitochondrial bioenergetics.

## Interconnected physiological function

Until now we have been taught to understand the body through the function of its organ systems as distinct operational units. Whilst this has been and continues to be essential to diagnose and manage illness, it fails to appreciate and address the simple fact that these units consist of networks that function in a highly interconnected fashion; thus change in any part of the network affects all other functional components.

Largely as a means to simplify, classify and explain whole body complexity, textbooks still describe physiologic function predominantly from a large scale perspective, organized according to operational units like the cardiovascular or neurological systems and so forth. This limiting perspective fails to appreciate the importance of small scale network interactions as the drivers of large scale phenotypical appearances. Small scale physiological and molecular networks, through their interconnected interdependencies, thus contribute to normal and abnormal organ system function.

## Physiological networks

We focus on genes and their related transcriptional and proteomic networks. We examine how arousal/stress systems influence inflammatory networks and the bioenergetics networks of mitochondria as well as how inflammation and mitochondrial bioenergetics affect the arousal/stress systems.

## Genome, transcriptome, proteome, and metabolome networks

Rarely is a gene responsible for a particular disease outcome. Instead, health and disease outcomes result from the interaction of many genes, i.e., diseases arise from *genome* interactions (Noble, 2011). Goh et al. (2007) mapped the phenotypic appearance of *human disease*—the *disease phenome*—and the underlying *disease gene networks*—the *disease genome*. These maps reveal important genome linked diseases and have clarified how and why certain diseases frequently form clusters within the same person.

**Figure 2:**
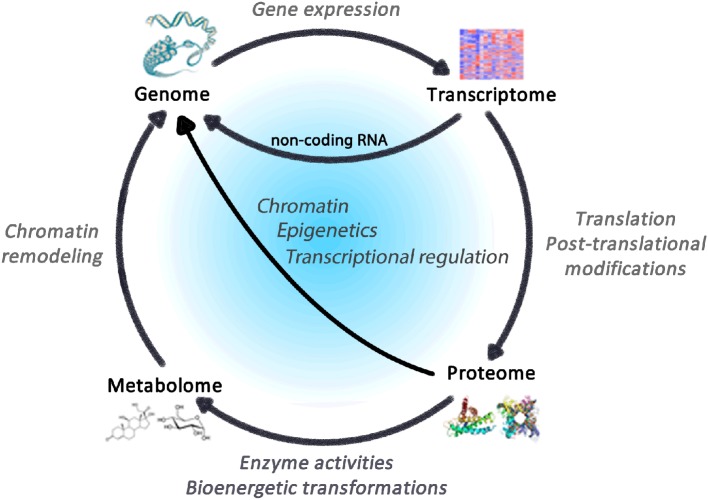
**Regulatory cycle linking the omics of life**. The genome comprises the totality of genes within an organism, which constitute the blueprint for the transcriptome, whose translation leads to proteins that accomplish enzymatic functions including bioenergetics transformations that consume and produce metabolites constituting the metabolome. In turn, gene transcripts, proteins and metabolites all impact expression genetic elements via dynamic processes subject to regulation.

The expression of the >25,000 genes that compose the human genome is regulated by several external factors that converge on gene regulatory pathways (Komili and Silver, 2008). These pathways entail specific transcription factors and chromatin-modifying epigenetic processes that activate or repress downstream genes (Portela and Esteller, 2010). This results in varying amounts of messenger RNA (mRNA) transcripts corresponding to different genes—the transcriptome. The transcriptome is processed [e.g., by alternative splicing (Luco et al., 2011)] and translated into proteins that collectively compose the proteome. Proteins then carry out all molecular and enzymatic activities, including those within mitochondria, that transform metabolic substrates into various metabolites (i.e., the metabolome) that also contribute to gene regulatory networks (Gut and Verdin, 2013) (Figure 2).

Thus, the genome, transcriptome, proteome and metabolome collectively give rise to cell functions and dysfunctions, underlying the development of pathophysiology. Common disease states are reflected at these different levels, including diabetes (Mootha et al., 2003; Wang et al., 2011), and other complex disease states such as Parkinson's disease, cancer, dementia and premature aging (Li et al., 2014). For example, the pathophysiological state leading to memory impairment is detectable in the metabolome and predicts later disease development (Mapstone et al., 2014). It is therefore relevant to understand how environmental factors influence these cellular networks by the activation of pleiotropic arousal systems and mitochondrial bioenergetics.

## Arousal and stress networks: getting under the skin

**Figure 3:**
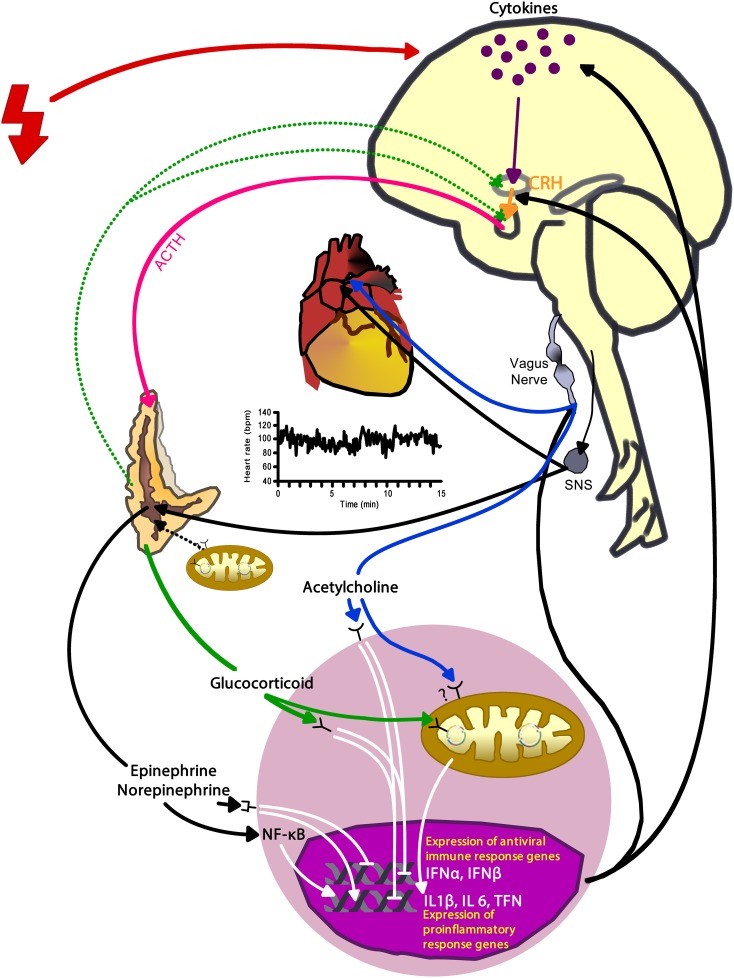
**Activation of arousal/stress systems—the autonomic nervous system and hypothalamic-pituitary-adrenal axis—has complex interconnections among the heart, immune cells, mitochondria, and gene expression that can also have modulating effects on the arousal/stress system activation**. Red bolt depicts internal and external stressors. CRH, corticotropin releasing hormone; ACTH, adrenocorticotropin hormone; SNS, sympathetic nervous system; INFα, interferon-alpha; INFβ, interferon-beta; IL, interleukin; TNF, tumor necrosis factor.

The external environment influences health through the brain's perception of the situational demands and learned or experiential skills to manage them (Lazarus and Folkman, 1984). If a situation requires more than the perceived ability to cope, then the nervous system sets into action a series of events enabling the body to overcome the excessive need. The sympathetic adrenal-medullary (SAM) and the hypothalamic-pituitary-adrenal (HPA) axes activate to support the behaviors that the brain perceived necessary to succeed or survive in a given situation. The SAM-axis floods the body with norepinephrine and epinephrine through endocrine and neural pathways, while the HPA-axis elevates hormones such as the corticotropin releasing hormone, adrenocorticotropin hormone, and cortisol (Bennett et al., 2013b) (Figure 3). These neuroendocrine hormones have a wide array of effects throughout the body; thus, providing the functional link between the external environment and overall health via the internal response to the perceived challenges or threats.

Of particular importance is how these neuroendocrine mediators impact immune cell function; influencing gene regulation and the cascade of transcriptome, proteome and metabolome function. For example, epinephrine and norepinephrine promote nuclear factor-kappa B (NF-κB) activation (Black, 2002; Bierhaus et al., 2003). NF-κB is a transcription factor that regulates gene expression of several proinflammatory mediators, such as IL-6 and IL-8, and enhances inflammation (Bierhaus et al., 2003). Cortisol can inhibit immune cell activity by binding to glucocorticoid receptors; this process inhibits activation and release of proinflammatory cytokines via inhibition of NF-κB (Barnes, 1998). However, chronic stress can lead to hippocampal damage and HPA axis dysregulation resulting in uncontrolled cortisol production (Sapolsky et al., 1985); immune cells downregulate expression of glucocorticoid receptors when exposed chronically to cortisol (Webster et al., 2002). As a result, chronic stress exposure can increase inflammation due to unregulated immune cells producing proinflammatory cytokines. Acetylcholine, the neurotransmitter of the parasympathetic nervous system (PNS), can decrease NF-κB activity via nicotinic acetylcholinergic receptors; resulting in reduced immune cell activity (Tracey, 2007). Thus, chronic stress/arousal leads to increased inflammation via diminished sensitivity to cortisol and excessive activation of the sympathetic nervous system (SNS) which suppresses the PNS and acetylcholine release.

It is through these arousal and stress responses that the external environment influences the internal physiological networks. Chronically, activation of the SAM and HPA-axes as well as elevated inflammation is linked to non-communicable diseases like cardiovascular disease, type 2 diabetes, depression, and osteoporosis (Bennett et al., 2013a). Chronic activation of the SAM and HPA-axes leads to tonic elevation in basal state and a loss of the ability to augment stress responses; leading to a loss in the network variability (and complexity of variability) and linked to disease, poor prognosis, and possibly accelerated aging (Bennett et al., 2013a).

Immunosenescence, the natural decline in variability of the adaptive immune system and increased activity of innate immunity (i.e., inflammation), occurs as the immune system ages (Franceschi et al., 2000; Fulop et al., 2010). For example, compromised cellular control of multiple *herpesviruses* has been linked to elevated inflammation (Bennett et al., 2012); suggesting that reduced adaptability of cellular immunity can fuel systemic inflammatory mediators. Elevated inflammation has independently been associated with age and frailty, morbidity, and mortality in elderly adults (Bennett et al., 2013a). The arousal and stress systems modify gene expression and cell functionality and these changes influence performance of the stress systems including the autonomic nervous system. Thus, examining the performance of adaptive and innate immunity and arousal/stress systems may provide a better estimate of the body's overall loss in fractal complexity.

## Mitochondrial bioenergetics

Mitochondria are symbiotic organelles producing the majority of cellular energy required for normal function. In evolution, approximately 1.5 billion years ago, the ancestor of today's eukaryotic cell engulfed a bacterium with the capacity to use oxygen for energy production, which later evolved as mitochondria (Sagan, 1967). The symbiotic relationship that emerged, with a newly acquired ability to make large amount of energy through aerobic metabolism, was a critical point that enabled the evolution of complex genomes and life forms (Lane and Martin, 2010; Wallace, 2010). For this and other reasons, abnormal mitochondrial bioenergetics can cause death in infancy or severe multisystemic pediatric and adult diseases (Koopman et al., 2012). From this symbiotic relationship, cells have therefore acquired particular sensitivity to bioenergetics signals from mitochondria.

The maintenance of physiological parameters within homeostatic/homeokinetic ranges requires continuous flux of energy. The maintenance of cell membrane potential, gene expression, protein and hormone biosynthesis, secretion, heart and muscle contraction, digestion and breathing are all processes requiring constant energy input in the form of adenosine triphosphate (ATP). Because mitochondria are the major source of cellular ATP, they are understood to maintain health by enabling cellular functions, and by determining adaptive capacity (Manoli et al., 2007). Whereas in humans mitochondrial defects predominantly affect the same organs (e.g., heart, muscles, and the brain) that preferentially decline with aging (Wallace, 2005); in animal models the accumulation of mitochondrial DNA (mtDNA) mutations that erode mitochondrial function accelerates the biological aging process (Trifunovic et al., 2004; Kujoth et al., 2005; Safdar et al., 2011), indicating their broad physiological effects of relevance to the aging process.

Mitochondria are also endowed with the capacity to sense neuroendocrine stress mediators and to produce signals of adaptation (Picard et al., 2014a). For instance, the glucocorticoid receptor (Lee et al., 2013) as well as other steroid hormone receptors are present in mitochondria (Psarra and Sekeris, 2009). The receptor activation by stress hormones influences mtDNA gene expression for the energy-producing machinery (Psarra and Sekeris, 2009). In turn, when their function is altered, mitochondria release signals that trickle down to the nucleus to influence expression of nuclear genes. For example, the progressive increase in mtDNA mutation load in human cells leads to dose-response changes in gene expression profiles, or “transcriptional reprogramming” affecting the majority of the human genome (Picard et al., 2014b). In fact, likely as a result of mitochondria's role in the evolution of eukaryotic cells, gene expression is under the control of various metabolites derived from mitochondrial function (Wallace and Fan, 2010; Gut and Verdin, 2013). Mitochondria thus lie at the interface of the neuroendocrine and metabolic environment, and the plastic (epi) genome that influence the aging trajectories (Picard, 2011).

Mitochondria are also linked to immune system activation and inflammation (Koshiba et al., 2011; West et al., 2011). Because the mtDNA is of bacterial origin, it is recognized as foreign by the body if released. Its release during mitochondrial damage can engage the immune system to promote inflammation (Zhang et al., 2010; Shimada et al., 2012). More specifically, mtDNA release from mitochondria exposed to oxidative stress engages the inflammasome (Lu et al., 2014). But these proinflammatory pathways can be blocked by acetylcholine, underscoring the interplay of mitochondria and neuroendocrine factors in response to stress (Lu et al., 2014).

Mitochondrial dysfunction can also influence SAM and HPA-axes function more directly. For example, mtDNA mutations that impair mitochondrial energy production lead to hyperactivation of the SNS in response to mild physical stress, as evidenced by the excessive epinephrine and norepinephrine secretion (Jeppesen et al., 2009). In addition, mitochondrial oxidative stress due to genetic defects of the mitochondrial antioxidant system leads to adrenal cortex atrophy and hypocortisolemia (Meimaridou et al., 2012). Both major stress response systems, the SAM and HPA-axes, are therefore subject to modulation by mitochondrial bioenergetics.

In summary, mitochondria are linked to other physiological networks of adaptation via inflammatory processes, and the SAM and HPA-axes. Mitochondrial bioenergetics also contributes to immediate cellular adaptation by determining maximal energy capacity and long-term by influencing gene expression patterns. This organelle is therefore functionally positioned to contribute to the age-related physiological decline via different inter-related pathways.

## Physiological networks and their relation to health and disease

Whilst we understand a great deal about the individual networks, and have a reasonable yet incomplete appreciation of their interdependencies, we have a limited understanding how these system interactions relate to health and disease. Nevertheless, viewing health and disease as functional consequences of small scale network functions and dysfunctions—maintaining or crossing homeostatic/homeokinetic boundaries—demands a very different way of thinking and managing a person's complaint. Ideally clinicians would only “nudge” these networks along to maintain their physiological homeostatic/homeokinetic range—narrowing with age—to achieve an optimal balance between ever decreasing physiological complexity and ever increasing entropy (Figure 1). This concept has not yet been explored in the literature.

Clinicians usually have to make pragmatic decisions at the time of the consultation and rely on the large scale characteristics (or phenotypic appearances) of disease/s. Given that the human body functions as a multi-scale entity it would be useful to rely on easily obtainable large scale measure/s of the underlying small scale network dys/function. Along those lines, in the social sciences, the highly inclusive and subjective measure of self-rated health (SRH) has been validated as an effective predictor of future health outcomes including mortality (Benyamini, 2011). More interestingly, SRH is a more valid health indicator than the combination of several objective biomarkers and sophisticated subjective constructs (Picard et al., 2013). Such a large-scale physiological measure of a system's complexity would provide a true reflection of the person's current state (rather than being “another” surrogate).

The measurement of variability of physiological parameters provides an indication of the overall function of the person. For example, Goldberger (1996) showed that the sinus wave pattern of heart rate variability (HRV) is associated with severe system dysfunction, and Bravi et al. (2012) demonstrated that the onset of sepsis in immune compromised patients is preceded by a progressive loss of inter beat HRV starting 60 h before its clinical diagnosis. We also have good evidence that specific medical conditions are associated with changes in their respective variability characteristics, like Parkinson's and Huntington's disease, Cushing's disease, diabetes, mental illness etc. (Sturmberg and West, 2013).

## Variability changes across the life span—the trajectory of decline with and without overt disease

Most aging people manage life well in spite of declining physiological stability. Clinically we see people age at different rates and in different patterns that can be categorized as symmetrical, where loss of function in all networks results in generalized frailty, or asymmetrical, where loss in one particular network—heart, kidneys, vision or memory—precedes that of all others.

Physiological functionality occurs within a homeostatic/homeokinetic range. These ranges of variability have not yet been clearly defined for healthy young volunteers, and are even less quantified regarding the changing patterns of homeostatic/homeokinetic ranges during growth and development or aging. Bioenergetic function, indexed by the body's maximal oxygen consumption capacity, experiences a steady decline with aging, which may underlie differential “pathways to aging” (Picard, 2011). Clinical experience shows that homeostatic/homeokinetic ranges are markedly different in the elderly, e.g., lowering blood pressure to “young adult” normal ranges is very frequently associated with lethargy, reduced cognitive function and falls secondary to cerebral hypo-perfusion (Mallery et al., 2014; Mossello et al., 2015; Sabayan and Westendorp, 2015)—stiffer vessels require a higher pressure to deliver the same amount of blood/oxygen, and older people with diabetes often experience hypoglycemic symptoms with even “high normal young adult” blood sugar readings (Lipska et al., 2014). These observations would indicate that homeostatic/homeokinetic ranges shift with aging to the right, or the width of the range may reduce.

## Variability and health and illness

**Figure 4:**
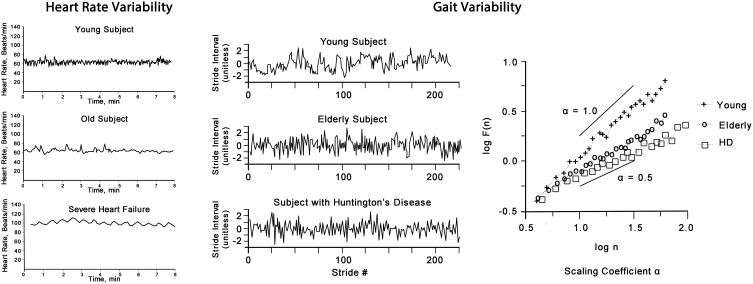
**Heart rate (left panel) shows the “physiological” decrease in HRV of an elderly person, however, it is greater than that of a person with severe heart failure (note: in AF HRV is greater than that of a health adult, not shown)**. Gait variability (right panel) shows the stride variability over time of a young, elderly and a person with Huntington's disease. The plot of F(n) by log(n) of this non-linear time-series of gait reveals the scaling exponent α which decreases with aging and is more pronounced in disease.

In health, variability measures show a high degree of variation and complexity, whereas illness is characterized by a variable losing its variability and complexity (Seely and Macklem, 2004). The magnitude of loss of variability correlates with the severity of the illness. Aging is associated with a general loss of complexity in physiological functioning involving all organ systems (Lipsitz and Goldberger, 1992; Vaillancourt and Newell, 2002). Whilst clinically healthy elderly show loss in variability, its degree is distinctively different to that seen in disease (Goldberger et al., 2002) as exemplified in Figure 4 for heart rate and gait variability.

## Heart rate variability

We believe that HRV may be utilized as an indicator of autonomic modulation, inter-organ coupling and overall system adaptability. High variability (including both degree and complexity of variation) is a sign of adaptability and health, whereas lower variability is a sign of inadequate adaptability and an indicator of physiological dysfunction. Such dysfunction can be caused by specific diseases, but also is an indicator of risk of adverse outcomes in otherwise clinically healthy people. Autonomic regulation, as reflected in HRV, is a vital mechanism for maintaining health (Pumprla et al., 2002). However, as Nicolini et al. (2012) pointed out, the concept of HRV may be viewed as a reflection of *autonomic modulation* of the *system wide adaptive processes* affecting all organ systems, where autonomic regulation results from multiple feedback loops of all other regulatory systems (Figure 3).

Beat to beat variation is regulated by parasympathetic synaptic acetylcholine and sympathetic synaptic norepinephrine release. Acetylcholine has a very short latency period and high reabsorption rate, and results in slowing of the heart rate. Norepinephrine in contrast is reabsorbed more slowly and results in increasing of the heart rate. The dynamics of the parasympathetic and sympathetic nervous system show different frequencies and can be identified in the HRV spectrum; higher frequency indicates parasympathetic, and low frequency sympathetic dominance (Nicolini et al., 2012).

Of note, the rapid control of heart rate is mediated by the parasympathetic pathway (Nicolini et al., 2012). In addition, the modulation of HRV through the autonomic nervous system itself is influenced by neuroendocrine feedback loops as shown in Figure 3. Adrenal stimulation by internal or external stressors increases adrenergic surges promoting proinflammatory activity. Disease processes result in the increase of inflammatory cytokines, which in turn activate the SAM and HPA-axes.

## The prognostic value of HRV

A fine-tuned highly adaptive system shows high degree of variation, highly complex variation, including fractal-like properties; and loss of degree and complexity of variation is associated with emerging pathology (Nicolini et al., 2012). In this context HRV has emerged as a robust and sensitive indicator of overall physiological functioning (Nicolini et al., 2012); HRV decreases with any disease, even those unrelated to heart disease (Tsuji et al., 1994; Dekker et al., 2000; Pumprla et al., 2002; Galluzzi et al., 2009; Stein et al., 2009; De Vilhena Toledo and Junqueira, 2010; Kemp et al., 2012; Nicolini et al., 2012; Madhavi and Ananth, 2013; Soares et al., 2013; Adlan et al., 2014; Harnod et al., 2014; Masel et al., 2014; De Couck and Gidron, 2013), and greater HRV decrease is a prognostic indicator of all-cause mortality (Tsuji et al., 1994; Mouton et al., 2012; Nicolini et al., 2012; De Couck and Gidron, 2013). Equally, HRV improvement is associated with improvement in disease states (Pumprla et al., 2002). Sometimes criticized for being overly sensitive, HRV, whilst not diagnostic of specific diseases, is a powerful indicator of altered health states (Madhavi and Ananth, 2013). Interestingly, a dose-response relationship exists between HRV and SRH, also considered to be a marker of general health (Jarczok et al., 2015).

The physiological changes associated with many diseases directly influence HRV modulating feedback pathways and explain Dekker and colleagues' proposition that low HRV is a general sign of poor health (although elevated HRV is seen in heart diseases like AF) (Dekker et al., 2000). HRV thus is a sensitive indicator of overall bodily function and could be utilized more in general medical practice both as a diagnostic and prognostic indicator.

Aging is a stochastic process and shows a high degree of individual variability after the age of 30 (Hayflick, 2000) due to a progressive loss of physiological reserve, and thus results in lower adaptability to changing internal and external challenges. This is observed also at the bioenergetics level, with an overall decline in maximal oxygen consumption capacity (VO_2max_) (Hawkins and Wiswell, 2003; Short et al., 2005; Kodama et al., 2009), which is dictated mainly by mitochondrial oxidative capacity (the ability of mitochondria to use oxygen for energy production) (Picard, 2011). Loss of mitochondrial oxidative capacity leads to decreased ability to burn oxygen to carbon dioxide and produce entropy (i.e., releasing it to the environment), thus inevitably leading to a rise in internal entropy content. This loss of oxygen consumption and entropy production affects both, the structure and function of the system, and results in the narrowing of the dynamic capacity to respond—the homeostatic/homeokinetic range becomes restricted. As hypothesized previously, fractal complex time series arise due to their proposed optimization of entropy production, and the loss of fractal complexity accompanies the loss of oxygen consumption and entropy production (Seely and Macklem, 2012). In summary, whilst ignoring the considerable complexities relating to standardized measurement of variability, the accumulating evidence points to the value of utilizing variability to track growth and development, to provide a measure of health, and to monitor the aging process.

Whilst we know that aging is associated with HRV decrease (Tsuji et al., 1994; Nicolini et al., 2012), reflecting the general decline in physiological adaptability, especially of the immune, bioenergetics and neuroendocrine components, we have so far no understanding how these changes interact and progress over time and might be driving disease development. To that end we propose a research agenda that explores aging patterns by following individuals over time. What is the relationship of HRV and gait variability? How do HRV changes relate to changes of SAM and HPA-axes or systemic inflammation and bioenergetics function, and how does disease exacerbation and recuperation change these markers? How do both degree and complexity of HRV measures change correlate with oxygen consumption? Such insights would enable doctors and patients to make better shared decisions about ongoing care—independent of the patient's specific diseases—that best reflects personal aspirations and medical possibilities, especially in the growing aging population.

What is required to harness this information is to create innovative bedside products using variability-derived prognostic information about individual patients, which demonstrate meaningful improvements to care in randomized controlled trials. This is an arduous, expensive and time-consuming process, and has been and remains a major barrier to bedside application. However, it can be done. An example of this approach is offered by the remarkable story of neonatal HRV monitoring; over a decade of research, several heart rate variability metrics along with clinical variables have been translated into a score reflecting a baby's risk of deterioration due to sepsis, which is monitored at the bedside as score (Toweill et al., 2000; Griffin and Moorman, 2001; Griffin et al., 2003, 2005a,b; Moorman et al., 2006). In a large 9 site multicenter randomized controlled clinical trial involving 3003 neonates with very low birth weight, simply displaying this score at the bedside led to a reduction of in-hospital mortality rate from 10.2 to 8.1% (*p* = 0.04) (Moorman et al., 2011). This reduction was particularly noteworthy given the fact that monitoring was un-protocolled, namely individual clinician's response was left up to their clinical judgment. This commercialization and randomized controlled evaluation is what is necessary to transform research into improvements in care.

## Conclusion

The human organism consists of a series of interdependent complex systems whose structure and function show high variability and demonstrate multi-scale self-similarity (fractal patterns). The “omics,” arousal/stress, immune, and bioenergetics systems and their multiplicative interconnected responses to perturbations regulate body function and show varying patterns across the trajectory of life—growth from birth through adolescence, plateauing in young adulthood and a steady decline throughout aging. They show high levels of variability, and higher variability is associated with better health across the lifespan. Aging involves the loss of system complexity and variability at multiple scales and these patterns are exacerbated in disease.

The current approach to understanding disease as resulting from perturbations of individual organ systems is enormously useful, but fails in the appreciation and study of the interconnected whole system, its complexity, adaptability and overall health of the human organism. Emerging evidence suggests that the organism's network interconnectedness and complexity can be described by the degree and complexity of biologic rhythm variability. The interconnected relationships of the various systems described point to HRV reflecting a global measure of an individual's current functional health state. Recognizing the theoretical nature of our argument, this is a call for the empirical examination of HRV's role in health, aging, and disease across the lifespan.

## Author contributions

JS conceived the paper; JS, JB, MP, and AS jointly drafted and critically reviewed the concepts presented in the paper. The authors approved the final version and agree to be accountable for all aspect of the work.

### Conflict of interest statement

Andrew J. E. Seely founded Therapeutic Monitoring Systems in order to commercialize patented Continuous Individualized Multiorgan Variability Analysis (CIMVA) technology, with the objective of delivering variability-directed clinical decision support to improve quality and efficiency of care. The authors declare that the research was conducted in the absence of any commercial or financial relationships that could be construed as a potential conflict of interest.
